# Supplementary material for: Converse flexoelectricity yields large piezoresponse force microscopy signals in non-piezoelectric materials
Source: Nat Commun. 2019 Mar 20;10:1266. doi: 10.1038/s41467-019-09266-y (PMC6427004; doi:10.1038/s41467-019-09266-y)
Supplement: Supplementary file 1 — Supplementary Information [file 41467_2019_9266_MOESM1_ESM.pdf]

Supplementary Information

Converse flexoelectricity yields large piezoresponse force microscopy signals in non-piezoelectric materials

Abdollahi et al.

Supplementary Note 1. Constitutive equations and numerical approximation

Following a linear continuum theory of piezoelectricity [1], augmented with flexoelectricity [2], the total electrical enthalpy of a dielectric solid possessing piezoelectricity and flexoelectricity is written as

$$\mathcal{H}(\varepsilon_{ij}, E_i, \nabla_l \varepsilon_{jk}) = \int_\Omega H(\varepsilon_{ij}, E_i, \nabla_l \varepsilon_{jk}) \, \mathrm{d}\Omega = \int_\Omega \frac{1}{2}\mathbb{C}_{ijkl}\varepsilon_{ij}\varepsilon_{kl} - e_{ikl}E_i\varepsilon_{kl}$$
$$-\mu_{ijkl}E_i\nabla_l\varepsilon_{jk} - \frac{1}{2}k_{ij}E_iE_j + \frac{1}{2}g_{ijklmn}\nabla_k\varepsilon_{ij}\nabla_n\varepsilon_{lm} \, \mathrm{d}\Omega, \tag{1}$$

where Ω is the solid domain, $\mathbb{C}$ is the fourth-rank tensor of elastic moduli, $\mathbf{e}$ is the third-rank tensor of piezoelectricity, $\mathbf{k}$ is the second-rank dielectric tensor, and $\mathbf{g}$ is the sixth-rank strain gradient elasticity tensor. The last term in Supplementary Eq. (1) is usually discarded, but it is important to guarantee the thermodynamic stability of the model in the presence of flexoelectricity [3, 4, 5]. The flexoelectric coupling between the strain gradient $\nabla\varepsilon$ and the electric field $\mathbf{E}$ is through the third term, where the fourth-rank flexoelectric tensor μ describes both direct and converse flexoelectric effects [6, 7]. The constitutive equations for the mechanical stress σ and the electric displacement $\mathbf{D}$ are derived from the electrical enthalpy density H as [8]

$$\sigma_{ij} = \hat{\sigma_{ij}} - \tilde{\sigma}_{ijk,k} = C_{ijkl}\varepsilon_{kl} - e_{kij}E_k + \mu_{lijk}E_{l,k} - g_{ijklmn}\varepsilon_{lm,nk}, \tag{2}$$

$$D_i = e_{ikl}\varepsilon_{kl} + \kappa_{ij}E_j + \mu_{ijkl}\varepsilon_{jk,l}, \tag{3}$$

where the third term in Supplementary Eq. (2) is the mechanical stress induced by converse flexoelectricity and the last term in Supplementary Eq. (3) is the polarization induced by direct flexoelectricity. The usual stress $\hat{\sigma}$, the higher-order (hyper) stress $\tilde{\sigma}$ and the electric displacement $\mathbf{D}$ are given by

$$\hat{\sigma}_{ij} = \frac{\partial H}{\partial \varepsilon_{ij}}, \qquad \tilde{\sigma}_{ijk} = \frac{\partial H}{\partial \varepsilon_{ij,k}}, \qquad \hat{D}_i = -\frac{\partial H}{\partial E_i}. \tag{4}$$

These equations are subject to appropriate electrical boundary conditions as

$$\phi = \bar{\phi} \quad on \quad \Gamma_\phi, \qquad D_in_i = -\omega \quad on \quad \Gamma_D, \tag{5}$$

where $\bar{\phi}$ and ω are the prescribed electric potential and surface charge density and $\Gamma_\phi \cup \Gamma_D = \partial\Omega$ is the boundary of the domain Ω with a unit normal n_i.
The mechanical boundary conditions in the form of displacements or traction $\mathbf{t}$ can be specified as below:

$$u_i = \overline{u}_i \quad \text{on} \quad \Gamma_u, \tag{6}$$

$$t_k = n_j(\hat{\sigma}_{jk} - \widetilde{\sigma}_{ijk,i}) - D_j(n_i\widetilde{\sigma}_{ijk}) - (D_pn_p)n_in_j\widetilde{\sigma}_{ijk} = \bar{t}_k \quad \text{on} \quad \Gamma_t, \tag{7}$$

where $\overline{u}_i$ and $\bar{t}_k$ are the prescribed mechanical displacements and tractions, $D_j = \partial_j - n_jD^n$ is the surface gradient operator, $D^n = n_k\partial_k$ is the normal gradient operator, $\Gamma_u \cup \Gamma_t = \partial\Omega$, and $\Gamma_u \cap \Gamma_t = \varnothing$. It is clear that the traction boundary condition in Supplementary Eq. (7) is affected by the higher-order stresses. In addition to these, the strain gradients result in other types of boundary conditions as [5]:

$$u_{i,j}n_j = \overline{v}_i \quad \text{on} \quad \Gamma_v, \tag{8}$$

$$n_jn_k\widetilde{\sigma}_{ijk} = \overline{r}_i \quad \text{on} \quad \Gamma_r, \tag{9}$$

where $\overline{v}$ is the prescribed normal derivative of displacement, $\overline{r}$ is the higher-order traction, $\Gamma_v \cup \Gamma_r = \partial\Omega$, and $\Gamma_v \cap \Gamma_r = \varnothing$. Here, we assume natural boundary conditions on Γ_v and Γ_r, i.e. $\overline{v} = \overline{\mathbf{r}} = \mathbf{0}$, and neglect edge forces arise from a non-smooth boundary [9].

The constitutive equations (2) and (3) in the presence of flexoelectricity are fourth-order which demand at least C^1 continuous basis functions for a direct Galerkin method. Therefore, the weak forms of the extended flexoelectric model cannot be discretized with standard finite elements. Following a recent work [8], we deal with the fourth-order nature of the partial differential equations by approximating displacements and electric potential using a meshfree method with smooth basis functions [10]. Meshfree methods provide an approximation to continuum field equations based on basis functions that do not rely on a mesh or its connectivity. Essentially, these methods allows us to determine a set of smooth basis functions $p^a(\mathbf{x})$, each localized around its corresponding node of the grid. We expand the continuum displacement and electric potential fields as

$$\mathbf{u}(\mathbf{x}) = \sum_{a=1}^{N} p^a(\mathbf{x})\mathbf{u}^a, \qquad \phi(\mathbf{x}) = \sum_{a=1}^{N} p^a(\mathbf{x})\phi^a, \tag{10}$$

and their derivatives as

$$\partial_j u_i = \textstyle\sum_{a=1}^{N} \partial_j p^a u_i^a, \tag{11}$$
$$\partial_j \partial_k u_i = \textstyle\sum_{a=1}^{N} \partial_j \partial_k p^a u_i^a, \tag{12}$$
$$\partial_j \phi = \textstyle\sum_{a=1}^{N} \partial_j p^a \phi^a, \tag{13}$$
$$\partial_j \partial_k \phi = \textstyle\sum_{a=1}^{N} \partial_j \partial_k p^a \phi^a, \tag{14}$$

where the first and second variations involve the gradient and Hessian of the LME basis functions, respectively. From now on, we omit the arguments of the basis functions for simplicity, i.e. $\mathbf{u} = \sum_{a=1}^{N} p^a \mathbf{u}^a$.

Introducing these expansions in Supplementary Eq. (1) and considering a 2D axisymmetric problem dealing with rotationally symmetric structures under axisymmetric loading, we obtain its discrete representation as

$$\begin{aligned}
\mathcal{H}(\mathbf{U},\phi) \quad = \quad & \pi \sum_{a,b} \mathbf{u}^{aT} \left(\int_{\Omega} \mathbf{B_u}(p^a) \mathbb{C} \mathbf{B_u}^T(p^b) \; r\mathrm{d}r\mathrm{d}z \right) \mathbf{u}^a \\
& + 2\pi \sum_{a,b} \mathbf{u}^{aT} \left(\int_{\Omega} \mathbf{H_u}(p^b) \mu^T \mathbf{B}_{\phi}^T(p^a) \; r\mathrm{d}r\mathrm{d}z \right) \phi^b \\
& + 2\pi \sum_{a,b} \mathbf{u}^{aT} \left(\int_{\Omega} \mathbf{B_u}(p^a) \mathbf{e}^T \mathbf{B}_{\phi}^T(p^b) \; r\mathrm{d}r\mathrm{d}z \right) \phi^b \\
& - \pi \sum_{a,b} \left(\int_{\Omega} \mathbf{B}_{\phi}(p^a) \mathbf{k} \mathbf{B}_{\phi}^T(p^b) \; r\mathrm{d}r\mathrm{d}z \right) \phi^a \phi^b \\
& + \pi \sum_{a,b} \mathbf{u}^{aT} \left(\int_{\Omega} \mathbf{H_s}(p^a) \mathbf{g} \mathbf{H_s}^T(p^b) \; r\mathrm{d}r\mathrm{d}z \right) \mathbf{u}^a,
\end{aligned} \tag{15}$$

where r and z are the cylindrical coordinates. The stiffness tensor $\mathbb{C}$, the dielectric tensor $\mathbf{k}$, the piezoelectric tensor $\mathbf{e}$, the flexoelectric tensor μ, and the strain-gradient tensor $\mathbf{g}$ are written in Voigt form as

$$\mathbb{C} = \begin{bmatrix} c_{11} & c_{12} & 0 & c_{12} \\ c_{12} & c_{11} & 0 & c_{12} \\ 0 & 0 & c_{44} & 0 \\ c_{12} & c_{12} & 0 & c_{11} \end{bmatrix}, \tag{16}$$

$$\mathbf{k} = \begin{bmatrix} \kappa_{11} & 0 \\ 0 & \kappa_{11} \end{bmatrix}, \tag{17}$$

$$\mathbf{e} = \begin{bmatrix} 0 & 0 & e_{15} & 0 \\ e_{31} & e_{33} & 0 & e_{31} \end{bmatrix}, \tag{18}$$

$$\mu = \begin{bmatrix} \mu_{11} & \mu_{13} & 0 & 0 & 0 & \mu_{44} & \mu_{13} \\ 0 & 0 & \mu_{44} & \mu_{13} & \mu_{11} & 0 & 0 \end{bmatrix}, \tag{19}$$

$$\mathbf{g} = l_1^2 \begin{bmatrix} c_{11} & c_{12} & 0 & c_{12} & 0 & 0 & 0 & 0 \\ c_{12} & c_{11} & 0 & c_{12} & 0 & 0 & 0 & 0 \\ 0 & 0 & c_{44} & 0 & 0 & 0 & 0 & 0 \\ c_{11} & c_{11} & 0 & c_{11} & 0 & 0 & 0 & 0 \\ 0 & 0 & 0 & 0 & c_{11} & 0 & 0 & 0 \\ 0 & 0 & 0 & 0 & 0 & 0 & c_{12} & c_{12} \\ 0 & 0 & 0 & 0 & 0 & c_{12} & 0 & 0 \\ 0 & 0 & 0 & 0 & 0 & c_{12} & 0 & 0 \end{bmatrix}, \tag{20}$$

where $c_{11} = E(1-\nu)/(1+\nu)(1-2\nu)$, $c_{12} = E\nu/(1+\nu)(1-2\nu)$, $c_{44} = c_{11}/2$. The longitudinal, transversal, and shear flexoelectric coefficients are represented by μ_{11}, μ_{13}, and μ_{44} respectively and k_{11} is the dielectric constant for an isotropic medium. The gradient operators $\mathbf{B_u}$ and $\mathbf{B}_\phi$ and the Hessian operators $\mathbf{H_u}$ and $\mathbf{H_s}$ for an axisymmetric case are written in Voigt form as

$$\mathbf{B_u} = \begin{bmatrix} \partial/\partial r & 0 & \partial/\partial z & 1/r \\ 0 & \partial/\partial z & \partial/\partial r & 0 \end{bmatrix}, \quad \mathbf{B}_\phi = \begin{bmatrix} \partial/\partial r & \partial/\partial z \end{bmatrix}, \tag{21}$$

$$\mathbf{H_u} = \begin{bmatrix} \frac{\partial^2}{\partial r^2} & 0 & \frac{\partial^2}{\partial r \partial z} & \frac{\partial^2}{\partial z \partial r} & 0 & \frac{\partial^2}{\partial z^2} & \frac{1}{r}\frac{\partial}{\partial r} - \frac{1}{r^2} \\ 0 & \frac{\partial^2}{\partial r \partial z} & \frac{\partial^2}{\partial r^2} & 0 & \frac{\partial^2}{\partial z^2} & \frac{\partial^2}{\partial z \partial r} & 0 \end{bmatrix}, \tag{22}$$

$$\mathbf{H_s} = \begin{bmatrix} \frac{\partial^2}{\partial r^2} & 0 & \frac{\partial^2}{\partial r \partial z} & \frac{\partial^2}{\partial z \partial r} & 0 & \frac{\partial^2}{\partial z^2} & \frac{1}{r}\frac{\partial}{\partial r} - \frac{1}{r^2} & \frac{1}{r}\frac{\partial}{\partial r} - \frac{1}{r^2} \\ 0 & \frac{\partial^2}{\partial r \partial z} & \frac{\partial^2}{\partial r^2} & 0 & \frac{\partial^2}{\partial z^2} & \frac{\partial^2}{\partial z \partial r} & 0 & 0 \end{bmatrix}. \tag{23}$$

The discrete algebraic equations for the equilibrium can be derived following the usual Galerkin procedure as

$$\begin{bmatrix} \mathbf{A_{UU}} & \mathbf{A_{U\phi}} \\ \mathbf{A_{\phi U}} & \mathbf{A_{\phi\phi}} \end{bmatrix} \begin{bmatrix} \mathbf{U} \\ \phi \end{bmatrix} = \begin{bmatrix} \mathbf{f_U} \\ \mathbf{f}_\phi \end{bmatrix}, \tag{24}$$

where the local contribution of each quadrature point to the matrix of system has the structure

$$\mathbf{A}^{ab}_{\mathbf{UU}} = \mathbf{B_u}(p^a)\mathbb{C}\mathbf{B_u}^T(p^b)r + \mathbf{H_s}(p^a)\mathbf{g}\mathbf{H_s}^T(p^b)r, \tag{25}$$

$$\mathbf{A}^{ab}_{\mathbf{U}\phi} = \mathbf{H_u}(p^a)\mu^T\mathbf{B}^T_\phi(p^b)r + \mathbf{B_u}(p^a)\mathbf{e}^T\mathbf{B}^T_\phi(p^b)r, \tag{26}$$

$$\mathbf{A}^{ab}_{\phi\mathbf{U}} = \mathbf{B}_\phi(p^b)\mu\mathbf{H_u}^T(p^a)r + \mathbf{B}_\phi(p^b)\mathbf{e}\mathbf{B_u}^T(p^a)r, \tag{27}$$

$$\mathbf{A}^{ab}_{\phi\phi} = -\mathbf{B}_\phi(p^a)\mathbf{k}\mathbf{B}^T_\phi(p^b)r, \tag{28}$$

$$\mathbf{f}_\phi = 0, \tag{29}$$

$$\mathbf{f_U} = \mathbf{0}, \tag{30}$$

where the derivatives of the basis functions are evaluated at the corresponding quadrature point.

Supplementary Note 2. Contact model

The Signorini-Hertz-Moreau model [12, 13] is written for a contact point pair ($\mathbf{x}$; $\mathbf{x}_0$) as:

$$g_n \leq 0, \quad \sigma_n = \mathbf{n} \cdot \sigma \cdot \mathbf{n} \leq 0, \quad g_n \sigma_n = 0, \quad \text{on } \Gamma_c \tag{31}$$

where $\mathbf{n}$ stands for the outward unit normal vector to the contact boundary Γ_c. The normal gap function g_n is given by

$$g_n = u_n + [\mathbf{x} - \mathbf{x}_0] \cdot \mathbf{n}, \tag{32}$$

where $u_n = \mathbf{u}\cdot\mathbf{n}$, $\mathbf{u}$ is the mechanical displacement, $\mathbf{x}$ is a point on the flat surface and $\mathbf{x}_0$ is the corresponding point of the rigid sphere which is coming into contact. The simplest method to enforce the non-penetration constraints in Supplementary Eq. (31) consists on transforming the constrained minimization problem into an unconstrained minimization problem by the penalty method, which adds an extra surface energy term over the contact boundary, penalizing deviation from the conditions in Supplementary Eq. (31), to the total energy in Supplementary Eq. (1) as [12]

$$\mathcal{H}_t = \mathcal{H} + \int_{\Gamma_c} \frac{1}{2}\beta \langle g_n \rangle^2 \, \mathrm{d}S, \tag{33}$$

where β is the penalty parameter and $\langle \bullet \rangle$ is the Macaulay brackets which returns the argument itself if the argument is positive, otherwise it returns zero (no contact).

The rotational symmetry of the spherical tip-sample contact allows us to employ a two-dimensional axisymmetric model, as depicted in Fig. 2(b) of the manuscript. The contact force F is then obtained as

$$F = 2\pi \int_0^a \beta \langle g_n \rangle r \, \mathrm{d}r, \tag{34}$$

where a is the contact radius, i.e. the distance of the farthest point from the symmetry axis where $\langle g_n \rangle \neq 0$. The discrete form of the total energy $\mathcal{H}_t$ in Supplementary Eq. (33) in then obtained as

$$\mathcal{H}_t(\mathbf{U}, \phi) = \mathcal{H}(\mathbf{U}, \phi) - \sum_a \mathbf{F}^a \cdot \mathbf{u}^a, \tag{35}$$

where $\mathcal{H}$ is given in Supplementary Eq. (15) and $\mathbf{F}^a$ is the contact force given by

$$\mathbf{F}^a = 2\pi \int_0^{R_c} \bar{\mathbf{t}} p^a \, r\mathrm{d}r, \tag{36}$$

where R_c is the contact radius and $\bar{\mathbf{t}}$ is the imposed mechanical traction due to contact, obtained as $\bar{\mathbf{t}} = -\beta\langle g_n \rangle \mathbf{n}$. The right-hand side contribution in Supplementary Eq. (30) then changes to $\mathbf{f}_\mathbf{U} = \bar{\mathbf{t}} p^a r$ and the total contact force $\mathbf{F}$ is obtained as $\mathbf{F} = \sum_a \mathbf{F}^a$.

To verify this contact model, we perform simulations for different given vertical positions of the tip and compute the magnitude of the resulting contact force. These results are compared with the classical Hertzian contact model which provides an analytical solution for the relationship between the indentation depth d, the tip radius R, the contact radius R_c, and the applied load F as [14]

$$d = \frac{R_c^2}{R} = \left(\frac{9F^2}{16{E^*}^2 R} \right)^{1/3}, \tag{37}$$

where E^* is the apparent Young's modulus. Assuming that the Young's modulus of the tip is noticeably higher than that of the sample, E^* is approximated as $E^* = E/(1-\nu^2)$, where E and ν are the Young's modulus and Poisson's ratio of the sample, respectively. Supplementary Figure 1 shows the results considering elastic parameters $E = 230$ GPa and $\nu = 0.3$, a tip radius of $R = 100$ nm, a domain length of $L = 2$ μm, a domain height of $H_d = 1$ μm and a value of 5×10^{17} Nm^{-3} for the penalty parameter β. A good agreement is observed between the simulation and the Hertzian contact model.

To perform the simulations of non-piezoelectric $SrTiO_3$ (STO), we consider the material parameters given in Ref. [11] and presented in Supplementary Table 1. The length-scale l_1 has been computed as 10 nm using

a formulation in Ref. [5]. Simulations are performed considering a tip radius of $R = 100$ nm, a domain length of $L = 2$ μm, a domain height of $H_d = 1$ μm and a tip voltage $V =$ -5 V. We first progressively indent (increase the vertical position of the tip), until reaching the desired force F in the absence of the tip voltage ($V = 0$). Then, in the second simulation, we apply the tip voltage V (see Eqs. (2) and (3) in the manuscript) and change the position of the tip until we obtain the same calculated force as in the first simulation. This change corresponds to the value of Δu_z. The effective piezoelectric coefficient is then calculated as $d_{33}^{\text{eff}} = -\Delta u_z/V$. We perform these simulations for different values of the applied force F.

Supplementary Note 3. Electrochemical strain

Another effect that can yield an electromechanical response is electrochemical strain [15]. The intense electric fields generated by the tip can sometimes attract ionic defects, such as oxygen vacancies, towards the contact region, thereby inducing a local swelling of the lattice. This effect is proportional to ionic conductivity and it should therefore be expected to increase with temperature [16]. In contrast, the converse flexoelectric coefficient is proportional to the dielectric constant, which in $SrTiO_3$ (STO) decreases with increasing temperature [17]. We have looked at the temperature dependence of the STO piezoresponse to distinguish both mechanisms. As can be seen in Supplementary Figure 2, the piezoresponse decreases as temperature increases, consistent with a flexoelectric origin. The ionic migration contribution to the electromechanical response was neglected due to the lack of hysteresis in the cycles of the remanent PFM response as a function of the applied V_{dc} field (Supplementary Figure 3). The absence of oxygen vacancies that could lead to ionic contribution to electromechanical response [18, 19] was also probed by XPS (Supplementary Figure 4).

Supplementary Note 4. XPS analysis of the $SrTiO_3$ Single Crystal

STO single crystals were analysed by XPS at room temperature and different water gas pressures. XPS experiments were performed at the Near Ambient Pressure XPS end station of the CIRCE beamline (BL-24) at the ALBA synchrotron radiation facility. The photon energy range at CIRCE is 100 eV-2000 eV. The end station is equipped with a Phoibos 150 NAP electron energy analyzer. The analyzer is provided with four differentially pumped stages connected by small apertures. A set of electrostatic lenses focuses the electrons though the apertures to maximize transmission. Such set-up allows keeping the detector in Ultra High Vacuum while the sample is at a maximum pressure of 20 mbar [20, 21]. The total electron energy resolution in the experimental conditions used for the high-resolution spectra was better than 0.3 eV. Further details about the system can be found elsewhere [22].

Surface oxygen vacancies can be monitored by checking the presence of shoulders in the low BE side of the $Ti^{4+}2p_{3/2}$ peak (corresponding to Ti^{4+}) due to reduced Ti species, i.e. Ti^{3+} (Supplementary Figure 4b). In our experiments the RH conditions change from vacuum to 10% and surface oxygen vacancies cannot be observed in the Ti $2p_{3/2}$ peak in any case. Since our samples were all previously exposed to atmospheric conditions and we did not perform any annealing treatment to create vacancies, we conclude that all oxygen vacancies close to the surface area within a penetration depth of several nm have been compensated before the experiments began. No differences in the shape of Ti $2p_{3/2}$ neither the Sr 3d peaks are observed before and after water exposure.

Supplementary Note 5. Topographical damage after electromechanical measurements

In order to account for possible electrochemical effects in the shape of permanent damage in the material produced by the application of an ac voltage by an Atomic Force Microscope (AFM) conductive tip, the topography of the sample was monitored before and after the experiments. Supplementary Figure 5 shows

that there is no relevant difference in sample topography before and after measuring the electromechanical response with a diamond coated tip.

Supplementary Note 6. Electromechanical response of TiO_2

The effective piezoelectric coefficient was measured for TiO_2 in the same conditions as for $SrTiO_3$. TiO2 shows a rutile structure as compared to the cubic STO structure and is known to also show a reasonable flexoelectric response, even its flexoelectric coefficient μ_{13}^{eff}=1.7 nCm^{-1} is reduced by a factor of 0.68 with respect of that of $SrTiO_3$. This lower flexoelectric performance leads to a reduction of the measured effective piezoelectric coefficient due to converse flexoelectric effects as expected. In this case, change of contact radius was achieved by using AFM tips with nominally different radius, and applying forces of the order of $F \approx 100$ nN for each of the measurements. In every measurement, the tip force was calibrated, tip radius was determined from SEM images after the measurements, and effective contact radius was calculated a posteriori using the Hertzian contact model. Measurements were done for each tip alternatively on each sample (Supplementary Figure 6).

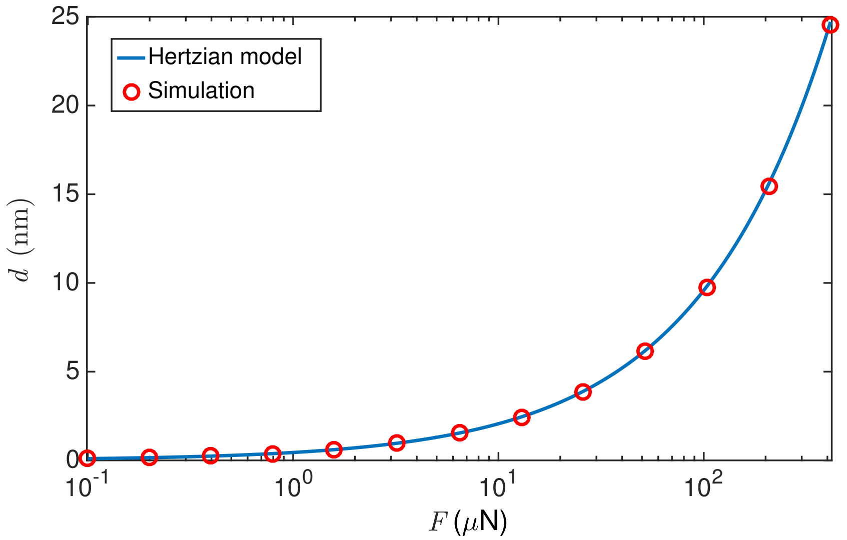

Supplementary Figure 1. Indentation depth d as a function of the applied force F for the contact of a rigid spherical tip with a flat surface. The results are obtained from the Hertzian contact model and simulations considering a tip radius of $R = 100$ nm. The Hertzian model provides an analytical solution as stated in Supplementary Eq. (37). The simulation results are obtained from the contact model presented in Supplementary Note 2. A good agreement is observed between the simulation and the Hertzian contact model.

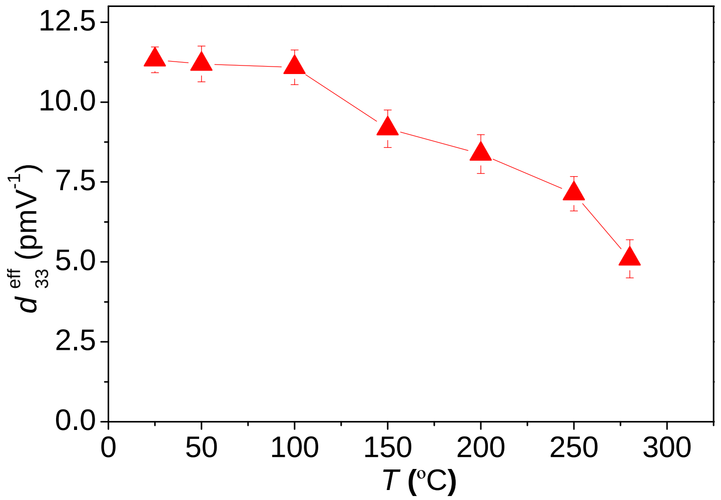

Supplementary Figure 2. Study of the evolution of the converse flexoelectricity as a function of temperature. The figure shows the measured effective piezoelectric coefficient as a function of temperature for the $SrTiO_3$ crystal, obtained with a Nanosensors CDT NCL tip with a cantilever of medium stiffness ($k \approx 2.8$ Nm^{-1}) coated with doped diamond. The temperature was gradually increased in a controlled way from room temperature to 275 °C in steps of 25 °C and 50 °C using a sample holder with a heater. The error bars correspond to the error of the linear fitting of the experimental data, which correlates the measured electromechanical amplitude of oscillation Δh with the V_{ac} applied voltage.

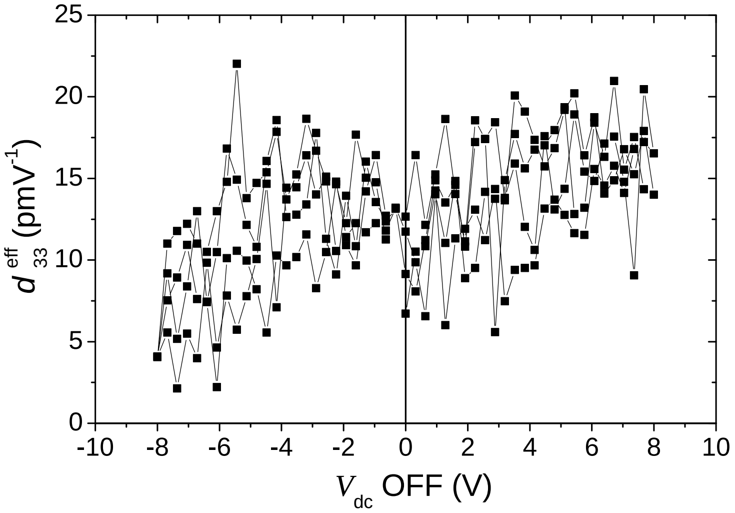

Supplementary Figure 3. Electromechanical response after removal of a V_{dc} voltage applied to the tip. The figure shows the hysteresis cycle of the electromechanical response obtained by measuring the remaining response after removal of a V_{dc} voltage applied to the tip. The lack of hysteresis confirms that the ionic migration contribution to the electromechanical response is not relevant for the effective d_{33} measurement.

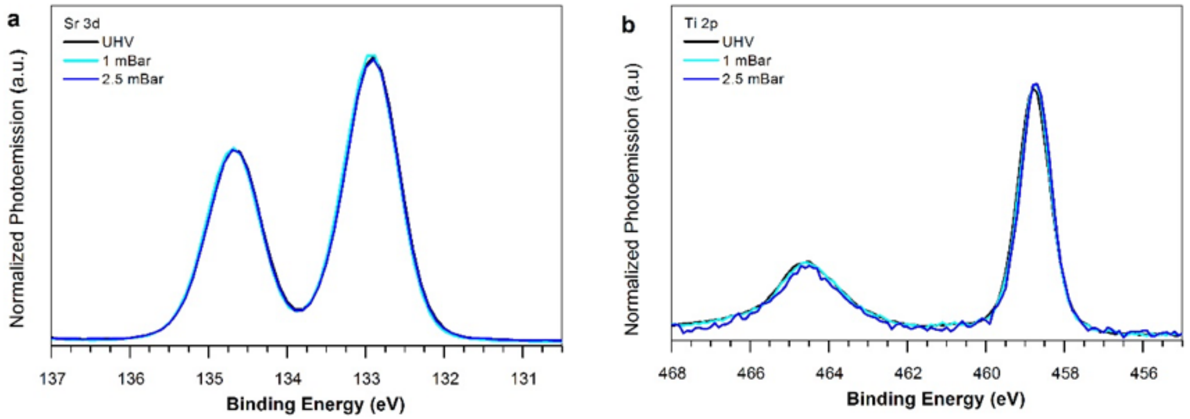

Supplementary Figure 4. X-ray Photoelectron Spectroscopy of the $SrTiO_3$ single crystal. **a** shows the X-ray Photoelectron Spectroscopy (XPS) spectra at the Sr 3d edge and **b** shows the XPS spectra at the Ti 2p edge. The doublet of peaks correspond to the Ti^{4+} oxidation state of the metallic ion. The lack of any shoulder to the pick associated with other oxidation state of Ti is a direct evidence of the absence of oxygen vacancies close to the surface.

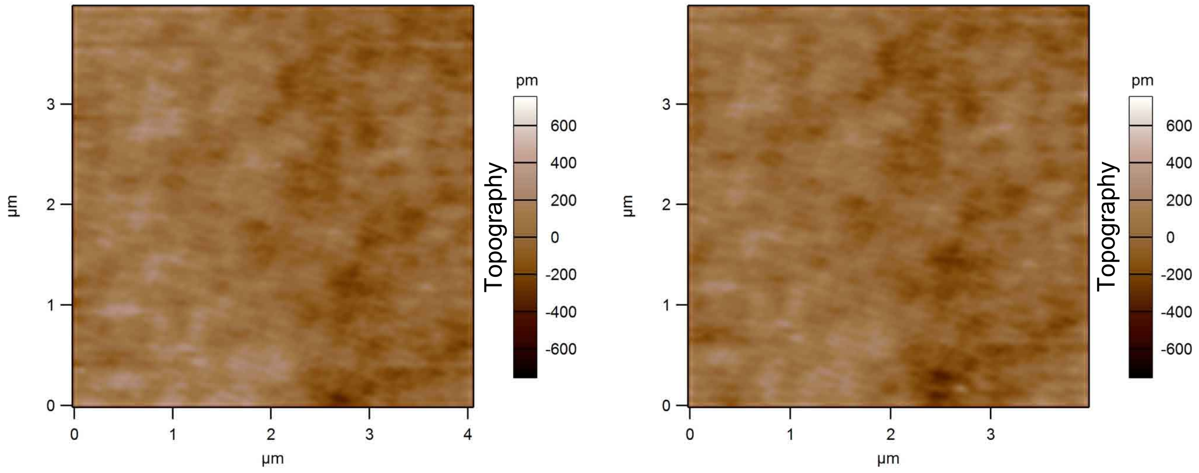

Supplementary Figure 5. Topographical study of possible electrochemical damages of the surface after the electromechanical measurements. **a** Atomic Force Microscopy (AFM) topography image in tapping mode of the surface topography of $SrTiO_3$ single crystal before the converse flexoelectric measurements, done with a Nanosensors PPP-CDT NCLR conductive diamond coated tip of medium stiffness ($k \approx 2.8$ Nm^{-1}). **b** AFM topography image after the converse flexoelectric measurement, showing no evidence of permanent damage of the surface due to i.e., electrochemical changes at the surface.

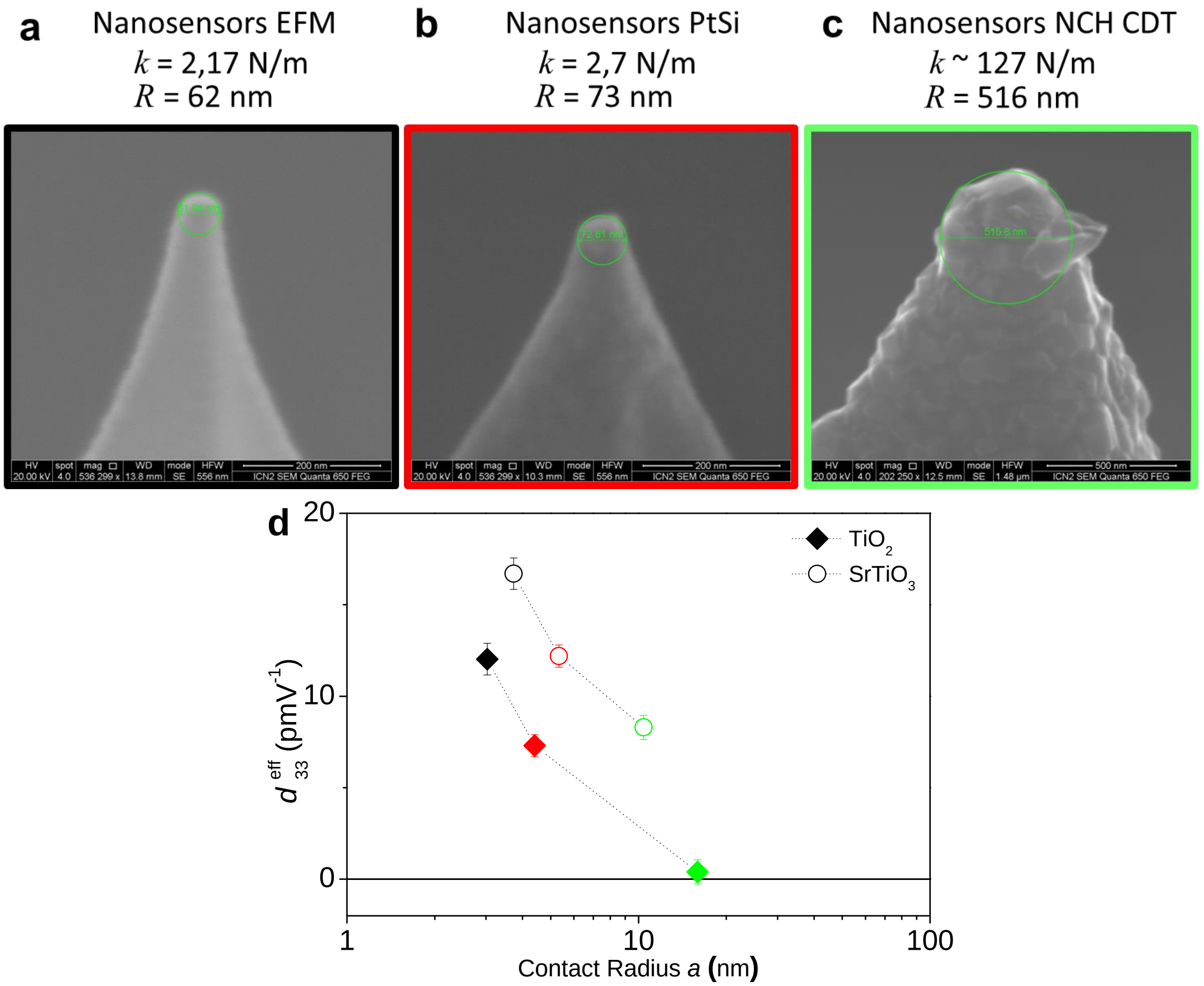

Supplementary Figure 6. Study of electromechanical response of TiO_2 as compared to $SrTiO_3$ under the use of tips with different radius. The radius of the tips was determined after the nanoscale electromechanical measurements from scanning electron microscopy (SEM) images of the used tips. **a** SEM image of a Nanosensor EFM tip of medium stiffness (k), showing the sharpest radius. **b** SEM image of Nanosensor PtSi tip also with a medium stiffness k. **c** SEM image of a Nanosensor NCH CDT diamond coated tip, with high stiffness and big tip radius. **d** Effective electromechanical response of TiO_2 (full diamonds) and $SrTiO_3$ (empty circles) as a function of the contact radius a. In this case, the different experimental contact radius were obtained by varying the tip radius instead of varying the force. The experimental contact radius a is calculated from the tip radius and the applied force following the Hertz contact model as stated in Supplementary Eq. (37). Under the same applied force, the contact radius will scale with the tip radius. The black points corresponding to the smaller contact radius are obtained from measurements performed with the sharper tip as shown in **a**, red points are obtained from measurements with the tip shown in **b** and finally green points correspond to measurements with the tip shown in **c**. The error bars correspond to the error of the linear fitting of the experimental data, which correlates the measured electromechanical amplitude of oscillation Δh with the V_{ac} applied voltage.

Supplementary Table 1. Material parameters for $SrTiO_3$

E	ν	μ_{11}	μ_{13}	μ_{44}	κ_{11}
230 GPa	0.3	7 nCm^{-1}	0.2 nCm^{-1}	5.8 nCm^{-1}	3 $nC(Vm)^{-1}$

Supplementary References

[1] Abdollahi, A. & Arias, I. Phase-field modeling of crack propagation in piezoelectric and ferroelectric materials with different electro-mechanical crack conditions. *J. Mech. Phys. Solids* **60**, 2100 (2012).

[2] Catalan, G., Sinnamon, L. J. & Gregg, J. M. The effect of flexoelectricity on the dielectric properties of inhomogeneously strained ferroelectric thin films. *J. Phys.: Cond. Matter* **16**, 2253 (2004).

[3] Maranganti, R., Sharma, N. D. & Sharma, P. Electromechanical coupling in nonpiezoelectric materials due to nanoscale nonlocal size effects: Green's function solutions and embedded inclusions. *Phys. Rev. B* **74**, 014110 (2006).

[4] Eliseev, A. E., Morozovska, A. N., Glinchuk, M. D. & Blinc, R. Spontaneous flexoelectric/flexomagnetic effect in nanoferroics. *Phys. Rev. B* **79**, 165433 (2009).

[5] Mao, S. & Prashant, P. K. Insights into flexoelectric solids from strain-gradient elasticity. *J. Appl. Mech.* **81**, 081004 (2014).

[6] Sharma, N. D., Landis, C. M. & Sharma, P. Piezoelectric thin-film superlattices without using piezoelectric materials. *J. Appl. Phys.* **108**, 024304 (2010).

[7] Sharma, N. D., Landis, C. & Sharma, P. Erratum: Piezoelectric thin-film super-lattices without using piezoelectric materials [j. appl. phys. 108, 024304 (2010)]. *J. Appl. Phys.* **111**, 059901 (2012).

[8] Abdollahi, A., Peco, C., Millán, D., Arroyo, M. & Arias, I. Computational evaluation of the flexoelectric effect in dielectric solids. *J. Appl. Phys.* **116**, 093502 (2014).

[9] Mindlin, R. Micro-structure in linear elasticity. *Arch. Rational Mech. Anal.* **16**, 51–78 (1964).

[10] Arroyo, M. & Ortiz, M. Local maximum-entropy approximation schemes: a seamless bridge between finite elements and meshfree methods. *Int. J. Numer. Meth. Eng.* **65**, 2167–2202 (2006).

[11] Zubko, P., Catalan, G., Buckley, A., Welche, P. R. L. & Scott, J. F. Erratum: Strain-gradient-induced polarization in $SrTiO_3$ single crystals [phys. rev. lett. 99, 167601 (2007)]. *Phys. Rev. Lett.* **100**, 199906 (2008).

[12] Wriggers, P. *Computational contact mechanics* (Springer Berlin Heidelberg, 2006).

[13] Yastrebov, V. & Breitkopf, P. *Numerical Methods in Contact Mechanics* (John Wiley and Sons, 2013).

[14] Johnson, K. L. *Contact Mechanics* (Cambridge University Press, 1985).

[15] Morozovska, A. N., Eliseev, E. A., Balke, N. & Kalinin, S. V. Local probing of ionic diffusion by electrochemical strain microscopy: Spatial resolution and signal formation mechanisms. *J. Appl. Phys.* **108**, 053712 (2010).

[16] Morozovska, A. N., Eliseev, E. A., Svechnikov, G. S. & Kalinin, S. V. Nanoscale electromechanics of paraelectric materials with mobile charges: Size effects and nonlinearity of electromechanical response of $srtio_3$ films. *Phys. Rev. B* **84**, 045402 (2011).

[17] Zubko, P., Catalan, G., Buckley, A., Welche, P. R. L. & Scott, J. F. Strain-gradient-induced polarization in SrTiO3 single crystals. *Phys. Rev. Lett.* **99**, 167601 (2007).

[18] Hanzig, J. *et al.* Migration-induced field-stabilized polar phase in strontium titanate single crystals at room temperature. *Phys. Rev. B* **88**, 024104 (2013).

[19] Khanbabaee, B. *et al.* Large piezoelectricity in electric-field modified single crystals of srtio3. *Appl. Phys. Lett.* **109**, 222901 (2016).

[20] Bluhm, H. Photoelectron spectroscopy of surfaces under humid conditions. *J. Elec. Spect. Rel. Phenom.* **177**, 71 – 84 (2010).

[21] Starr, D. E., Liu, Z., Hävecker, M., Knop-Gericke, A. & Bluhm, H. Investigation of solid/vapor interfaces using ambient pressure X-ray photoelectron spectroscopy. *Chem. Soc. Rev.* **42**, 5833–5857 (2013).

[22] Pérez-Dieste, V. *et al.* Near ambient pressure XPS at ALBA. *J. Phys.: Conference Series* **425**, 072023 (2013).
